# Supplementary material for: Yellow fever surveillance suggests zoonotic and anthroponotic emergent potential
Source: Commun Biol. 2022 Jun 2;5:530. doi: 10.1038/s42003-022-03492-9 (PMC9163115; doi:10.1038/s42003-022-03492-9)
Supplement: Supplementary file 2 — Supplementary Information [file 42003_2022_3492_MOESM2_ESM.pdf]

# Supplementary Information

## Yellow fever surveillance suggests zoonotic and anthroponotic emergent potential

Alisa Aliaga-Samanez[1], Raimundo Real[1,2], Marina Segura[3], Carlos Marfil-Daza[1], Jesús Olivero[1,2]

[1]Grupo de Biogeografía, Diversidad y Conservación, Departamento de Biología Animal, Facultad de Ciencias, Universidad de Málaga, 29071 Malaga, Spain

[2]Instituto IBYDA, Centro de Experimentación Grice-Hutchinson, Malaga, Spain

[3]Centro de Vacunación Internacional, Ministerio de Sanidad, Consumo y Bienestar Social, Estación Marítima, Recinto del Puerto, Muelle 3, 29001 Malaga, Spain

**The following sections include:**

- **Supplementary Figs. 1 – 2**
- **Supplementary Tables 1 – 3**
- **Supplementary Methods**

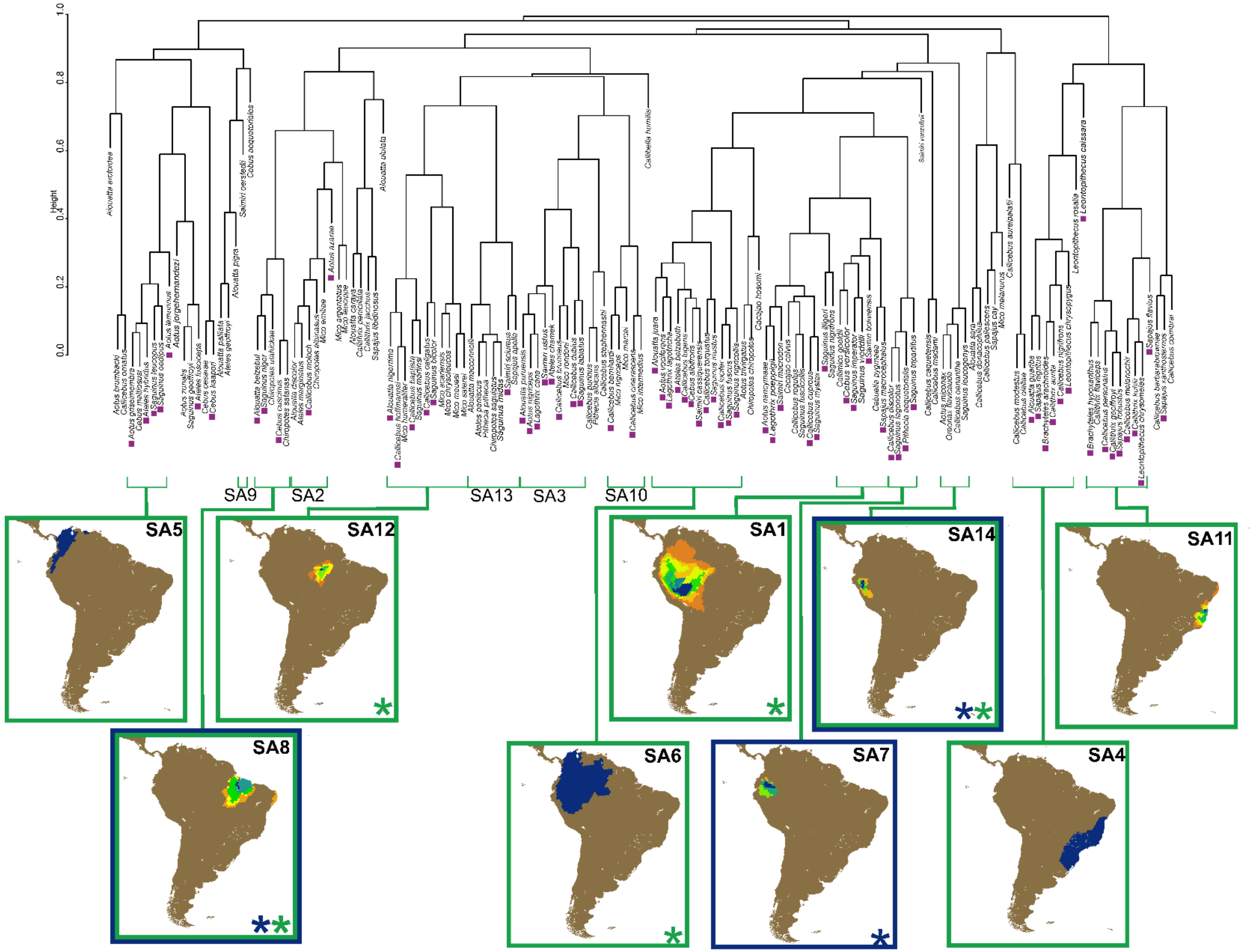


**Supplementary Fig. 1:** Classification dendrograms of primate distributions in America. Blue rectangles: chorotypes significantly related to the distribution of the late 20th-century yellow fever cases according to a forward-stepwise logistic regression. Green rectangles: chorotypes significantly related only to the distribution of the 21st-century cases. Chorotypes that were finally included in disease models are highlighted with a blue asterisk for the late 20th century, and with a green asterisk for the 21st century. American chorotype names are coded as SA1 to SA14. Violet square: species belonging to chorotypes significantly related to the yellow fever distribution (degree of membership ≥ 0,5).

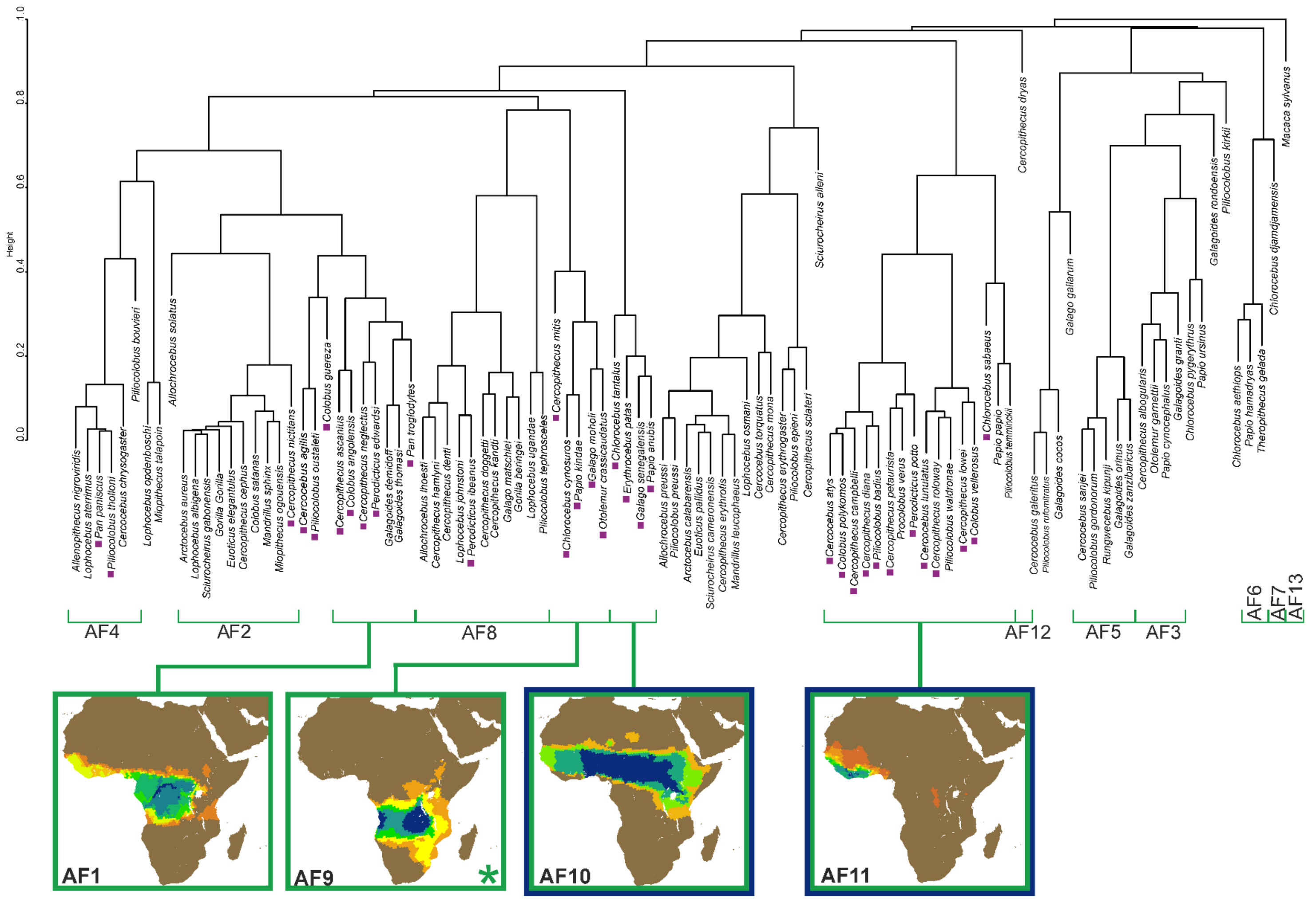


**Supplementary Fig. 2:** Classification dendrograms of primate distributions in Africa. Blue rectangles: chorotypes significantly related to the distribution of the late 20th-century yellow fever cases according to a forward-stepwise logistic regression. Green rectangles: chorotypes significantly related only to the distribution of the 21st-century cases. Chorotypes that were finally included in disease models are highlighted with a blue asterisk for the late 20th century, and with a green asterisk for the 21st century. American chorotype names are coded as AF1 to AF13. Violet square: species belonging to chorotypes significantly related to the yellow fever distribution (degree of membership ≥ 0,5).

**Supplementary Table 1:** Non-human primate genera included in the chorotypes that were significantly related to the distribution of yellow fever cases in the late 20th century and the early 21st century. SA: South-American chorotype; AF: African chorotype. See the classification dendrograms of primate distributions in South-America and Africa in Supplementary Fig. 1 and 2.

| Model | Chorotype | Genera |
|---|---|---|
| **20th century** | SA7 | *Callicebus*, *Pithecia*, *Saguinus* |
| | SA8 | *Alouatta*, *Cebus*, *Chiropotes* |
| | SA14 | *Saguinus*, *Brachyteles*, *Callithrix*, *Callicebus*, *Leontophitecus* |
| | AF10 | *Chlorocebus*, *Erythrocebus*, *Galago*, *Papio* |
| | AF11 | *Colobus*, *Cercopithecus*, *Piliocolobus*, *Cercocebus*, *Perodicticus*, *Procolobus* |
| **21st century*** | SA1 | *Callimico*, *Cebus*, *Callithrix*, *Saguinus*, *Saimiri*, *Sapajus* |
| | SA4 | *Alouatta*, *Sapajus*, *Brachyteles*, *Callithrix*, *Callicebus*, *Leontophitecus* |
| | SA5 | *Aotus*, *Cebus*, *Ateles*, *Saguinus* |
| | SA6 | *Alouatta*, *Aotus*, *Ateles*, *Callicebus*, *Saguinus*, *Saimiri* |
| | SA11 | *Brachyteles*, *Callithrix*, *Callicebus*, *Sapajus*, *Leontopithecus* |
| | SA12 | *Alouatta*, *Callicebus*, *Mico*, *Saguinus* |
| | AF1 | *Cercopithecus*, *Colobus*, *Perodicticus*, *Galagoides, Pan* |
| | AF9 | *Cercopithecus*, *Chlorocebus*, *Galago*, *Otolemur*, *Papio* |

(*) All chorotypes in the 20th-century model are also included in the 21st-century model. Only additional chorotypes are shown here.

**Supplementary Table 2:** Baseline disease model logit equations (i.e., linear combinations of predictor variables that form part of the logistic-regression equations). B: variable coefficient; SE: standard error; W: Wald parameter; DF: degrees of freedom; S: statistical significance. Variable codes as in Supplementary Table 1.

| **20th-century model** | | | | | |
|---|---|---|---|---|---|
| **Variable** | **B** | **SE** | **W** | **DF** | **S** |
| *Bio12* | $0.096 \times 10^{-3}$ | $0.104 \times 10^{-3}$ | 0.844 | 1 | 0.358 |
| *Dist_pop* | $-0.034 \times 10^{-3}$ | $0.005 \times 10^{-3}$ | 55.15 | 1 | $0.112 \times 10^{-14}$ |
| *Slope* | 0.121 | 0.048 | 6.492 | 1 | 0.011 |
| *DeXS* | -2.784 | 0.947 | 8.636 | 1 | 0.003 |
| *Famerica* | 9.282 | 0.559 | 275.365 | 1 | $0.769 \times 10^{-63}$ |
| *Fafrica* | 6.805 | 0.422 | 260.479 | 1 | $0.135 \times 10^{-59}$ |
| *SA7* | 0.714 | 0.132 | 29.274 | 1 | $0.628 \times 10^{-9}$ |
| *SA8* | 0.334 | 0.084 | 16.01 | 1 | $0.063 \times 10^{-3}$ |
| *SA14* | 0.542 | 0.187 | 8.416 | 1 | 0.004 |
| *Constant* | -6.959 | 0.388 | 321.032 | 1 | $0.863 \times 10^{-73}$ |
| *Goodness of fit* | $\chi^2 = 4.214$; $n = 10$ bins; $p=0.837$ | | | | |

| **21st-century model** | | | | | |
|---|---|---|---|---|---|
| **Variable** | **B** | **SE** | **W** | **DF** | **S** |
| *Y-20th century* | 0.012 | 0.059 | 0.045 | 1 | 0.833 |
| *Bio12* | $0.161 \times 10^{-3}$ | $0.019 \times 10^{-3}$ | 2.165 | 1 | 0.141 |
| *Bio5* | 0.016 | 0.002 | 44.564 | 1 | $0.246 \times 10^{-12}$ |
| *Bio6* | 0.007 | 0.002 | 7.633 | 1 | 0.006 |
| *Dist_pop* | $-0.026 \times 10^{-3}$ | $0.003 \times 10^{-3}$ | 60.015 | 1 | $0.941 \times 10^{-16}$ |
| *Elev* | 0.001 | $0.219 \times 10^{-3}$ | 29.283 | 1 | $0.625 \times 10^{-9}$ |
| *DeXS* | -2.759 | 0.688 | 16.068 | 1 | $0.061 \times 10^{-3}$ |
| *Famerica* | 7.983 | 0.605 | 174.096 | 1 | $0.943 \times 10^{-42}$ |
| *Fafrica* | 5.479 | 0.46 | 141.984 | 1 | $0.981 \times 10^{-34}$ |
| *SA1* | 0.26 | 0.047 | 31.12 | 1 | $0.243 \times 10^{-9}$ |
| *SA6* | 0.127 | 0.036 | 12.215 | 1 | $0.474 \times 10^{-3}$ |
| *SA8* | 0.625 | 0.084 | 55.567 | 1 | $0.903 \times 10^{-15}$ |
| *SA12* | 0.313 | 0.108 | 8.422 | 1 | 0.004 |
| *SA14* | 0.548 | 0.194 | 7.95 | 1 | 0.005 |
| *AF9* | 0.257 | 0.075 | 11.658 | 1 | 0.001 |
| *Constant* | -12.354 | 1.349 | 83.913 | 1 | $0.517 \times 10^{-21}$ |
| *Goodness of fit* | $\chi^2 = 6.499$; $n = 10$ bins; $p=0.592$ | | | | |

| **21st-century enhanced model** | | | | | |
|---|---|---|---|---|---|
| **Variable** | **B** | **SE** | **W** | **DF** | **S** |
| *Y-20th century* | 0.115 | 0.053 | 4.661 | 1 | 0.031 |
| *Bio12* | $0.202 \times 10^{-3}$ | $0.111 \times 10^{-3}$ | 3.303 | 1 | 0.069 |
| *Bio5* | 0.015 | 0.003 | 36.517 | 1 | $0.151 \times 10^{-10}$ |

| | | | | | |
|---|---|---|---|---|---|
| *Class 100* | -752.147 | 831.976 | 0.817 | 1 | 0.366 |
| *Class 110* | 2.285 | 0.662 | 11.906 | 1 | 0.001 |
| *Class 130* | -0.63 | 0.393 | 2.563 | 1 | 0.109 |
| *Class 200* | -12.12 | 5.11 | 5.626 | 1 | 0.018 |
| *Dist_pop* | $-0.023\times10^{-3}$ | $0.003\times10^{-3}$ | 50.083 | 1 | $0.147\times10^{-13}$ |
| *Elev* | 0.001 | $0.176\times10^{-3}$ | 26.835 | 1 | $0.222\times10^{-8}$ |
| *Forest loss* | 3.967 | 0.903 | 19.287 | 1 | $0.011\times10^{-3}$ |
| *Famerica* | 6.845 | 0.583 | 137.899 | 1 | $0.766\times10^{-33}$ |
| *Fafrica* | 4.820 | 0.449 | 115.32 | 1 | $0.670\times10^{-28}$ |
| *SA1* | 0.265 | 0.048 | 30.900 | 1 | $0.272\times10^{-9}$ |
| *SA6* | 0.146 | 0.038 | 14.917 | 1 | $0.112\times10^{-3}$ |
| *SA8* | 0.508 | 0.085 | 35.764 | 1 | $0.223\times10^{-10}$ |
| *SA12* | 0.337 | 0.109 | 9.560 | 1 | 0.002 |
| *SA14* | 0.463 | 0.196 | 5.576 | 1 | 0.018 |
| *AF9* | 0.310 | 0.074 | 17.348 | 1 | $0.031\times10^{-3}$ |
| *Constant* | -10.302 | 1.195 | 74.274 | 1 | $0.680\times10^{-19}$ |
| *Goodness of fit* | $\chi^2 = 10.818$; *n* = 10 bins; p=0.212 | | | | |

**Supplementary Table 3: Independent predictor variables considered for disease, vector, and transmission-risk modelling.** Some variables were used only in specific models: *20$^{th}$ century models; ** enhanced 21$^{st}$ century models; ***enhanced 21$^{st}$ century vector models; ****disease models.

| Factor | Code | Variable | Source |
|---|---|---|---|
| Climate | *Bio1* | Annual Mean Temperature | Chelsa (http://chelsa-climate.org) |
| | *Bio5* | Max Temperature of Warmest Month | |
| | *Bio6* | Min Temperature of Coldest Month | |
| | *Bio7* | Temperature Annual Range (Bio5-Bio6) | |
| | *Bio12* | Annual Precipitation | |
| | *Bio15* | Precipitation Seasonality (Coefficient of Variation) | |
| Human Concentration | *Pop_den* | Population density** | Administrative Centres & Populated Places shapefile at the Relational World Database II (RWDB2) updated in 2000 (http://www.fao.org/geonetwork) |
| | *Dist_pop* | Distance to populated places | |
| Infrastructures** | *Dist_road* | Distance to roads | Vector Map Level 0 at the Digital Chart of the World (DCW,http://worldmap.harvard.edu), updated in 2002 |
| | *Dist_rail* | Distance to rail-roads | |
| Livestock*** | *Buffaloes* | Density of buffaloes | FAO 2010(http://www.fao.org/livestock-systems/en/) |
| | *Poultry* | Density of poultry | |
| | *Goats* | Density of small ruminants (goats) | |
| | *Pigs* | Density of pigs | |
| | *Sheep* | Density of small ruminants (sheep) | |
| | *Cattle* | Density of cattle | |
| Topography | *Slope* | slope | From GTOPO30 (US Geological Survey 1996), using ArcGIS Desktop 10.3. |
| | *Elev* | Elevation | GTOPO30 (US Geological Survey 1996). |
| Hydrography | *Dist_riv* | Distance to rivers | Global Drainage Basin Database GDBD. Released Version 1.0: May 29, 2007 (http://www.cger.nies.go.jp/db/gdbd/gdbd_index_e.html). |
| Ecoregions* | *MedFWS* | Mediterranean Forest, Woodlands and Scrub | Terrestrial Ecoregions of the World: A New Map of Life on Earth: A new global map of terrestrial ecoregions provides an innovative tool for conserving biodiversity[1] |
| | *TrosubDBF* | Tropical and Subtropical Dry Broadleaf Forest | |
| | *TempCF* | Temperate Coniferous Forests | |
| | *TempBMF* | Temperate Broadleaf and Mixed Forests | |
| | *TrosubCF* | Tropical and Subtropical Coniferous Forests | |
| | *DeXS* | Deserts and Xeric Shrublands | |
| | *Mangro* | Mangroves | |
| | *TrosubMBF* | Tropical and Subtropical Moist Broadleaf Forest | |
| | *BorFT* | Boreal Forests/Taiga | |
| | *TrosubGSS* | Tropical and Subtropical Grasslands, Savannas and Shrublands | |
| | *TempGSS* | Temperate Grasslands, Savannas and Shrublands | |
| | *FloGS* | Flooded Grassland and Savannas | |
| | *MonGS* | Montane Grasslands and Shrublands | |
| | *Tundra* | Tundra | |

| | | | |
|---|---|---|---|
| Agriculture | *Class 11-14* | Croplands | GlobCover (GC) Land Cover version 2.3 database for 2009[2] |
| | *Class 20* | Mosaic Cropland (50-70%) / Vegetation (grassland, shrubland, forest) (20-50%)** | |
| | *Class 30* | Mosaic Vegetation (grassland, shrubland, forest) (50-70%) / Cropland (20-50%)** | |
| | *Equi_irrig* | Percentage of area equipped for irrigation*** | Global Map of Irrigation Areas (version 4.0.1) around the year 2000 (http://www.fao.org/nr/water) |
| Ecosystem Types** | *Class 40* | Closed to open (>15%) broadleaved evergreen and/or semi-deciduous forest (>5m) | GlobCover (GC) Land Cover version 2.3 database for 2009[2] |
| | *Class 50* | Closed (>40%) broadleaved deciduous forest (>5m) | |
| | *Class 60* | Open (15-40%) broadleaved deciduous forest (>5m) | |
| | *Class 70* | Closed (>40%) needleleaved evergreen forest (>5m) | |
| | *Class 90* | Open (15-40%) needleleaved deciduous or evergreen forest (>5m) | |
| | *Class 100* | Closed to open (>15%) mixed broadleaved and needleleaved forest (>5m) | |
| | *Class 110* | Mosaic Forest/Shrubland (50-70%) / Grassland (20-50%) | |
| | *Class 120* | Mosaic Grassland (50-70%) / Forest/Shrubland (20-50%) | |
| | *Class 130* | Closed to open (>15%) shrubland (<5m) | |
| | *Class 140* | Closed to open (>15%) grassland | |
| | *Class 150* | Sparse (>15%) vegetation (woody vegetation, shrubs, grassland) | |
| | *Class 160* | Closed (>40%) broadleaved semi-deciduous and/or evergreen forest regularly flooded - Saline water | |
| | *Class 170* | Closed (>40%) broadleaved semi-deciduous and/or evergreen forest regularly flooded - Saline water | |
| | *Class 180* | Closed to open (>15%) vegetation (grassland, shrubland, woody vegetation) on regularly flooded or waterlogged soil - Fresh, brackish or saline water | |
| | *Class 200* | Bare areas | |
| | *Class 220* | Permanent snow and ice | |
| Forest loss** | *Forest loss* | Non intact forest | High-Resolution Global Maps of $21^{st}$-Century Forest Cover Change[3] |
| Logit equation | *Y-20$^{th}$ century* | $20^{th}$-century-model logit equation | Linear combinations of predictor variables that form part of the logistic-regression equations |
| Spatial descriptors | *Fx* | Spatial trend, where “x” represents a continent | Linear combination of spatial variables derived from continental-scale trend surface analyses[4] |
| Primate chorotypes**** | *SAx* and *AFy* | Chorotype species richness. *SAx*: South-American chorotype “x” and *AFy*: African chorotype “y” | Supplementary Figs. 5-7[5] |

**Supplementary References:**


1. Olson DM, Dinerstein E, Wikramanayake E, Burgess ND, Powell G, Underwood E, et al. Terrestrial Ecoregions of the World: A New Map of Life on Earth: A new global map of terrestrial ecoregions provides an innovative tool for conserving biodiversity. Bioscience. 2001; 51: 933–938.
2. Bontemps S. GLOBCOVER 2009 Products Description and Validation Report. 2011.
3. Hansen MC. High-resolution global maps of $21^{st}$-century forest cover change. Science. 2013;342: 850–853
4. Legendre P. Spatial autocorrelation: Trouble or New Paradigm? Ecology. 1993;74: 1659–1673
5. Olivero J, Real R, Márquez AL. Fuzzy chorotypes as a conceptual tool to improve insight into biogeographic patterns. Syst Biol. 2011;60: 645–660. doi:10.1093/sysbio/syr026

## Supplementary methods

Methodological explanations for the variables and outputs shown in Fig. 4 of the main text. These variables and outputs are related to the following sections through letter codes (A – F).

### A. Independent variables: Environment

1. Preselection of environmental variables potentially influencing the distribution of the item to be modelled (i.e., a mosquito species, yellow fever cases). Variable sources and descriptions are shown in Supplementary Table 3.

### B. Independent variables: Space

1. Selection of the geographic context: A spatial independent variable was made for each continent (*x*).
2. Logistic regression: It provides a probability value ($P_x$), based on spatial descriptors, of the analysed item to occur in each hexagon:
   a. Dependent variable: Presences (1) and absences (0) of the item to be modelled.
   b. Initial independent variables: Spatial descriptors consisting on polynomial combinations of longitude (X) and latitude (Y) up to the third degree: X, Y, XY, $X^2$, $Y^2$, $X^2Y$, $XY^2$, $X^3$, and $Y^3$.
   c. Selection of independent variables: Conditional backward-stepwise procedure.
   d. Estimation of variable coefficients: machine-learning iterative process based on the maximum log-likelihood criterion.
   e. Model evaluation: Hosmer & Lemeshow's goodness of fit.

**SPSS syntax for the logistic regression**

```
LOGISTIC REGRESSION VARIABLES Dependent_variable
  /METHOD=BSTEP(COND) List_of_spatial_descriptors
  /SAVE=PRED
  /CRITERIA=PIN(.05) POUT(.10) ITERATE(20) CUT(.5).
```

3. A spatial independent variable is defined by the spatial favourability ($F_x$), where *x* represents a continent. $F_x$ provides a value of the degree to which spatial descriptors favour the occurrence of the analysed item in each hexagon.

$$F_x = \frac{P_x}{1-P_x} \Big/ \left(\frac{n_1}{n_0} + \frac{P_x}{1-P_x}\right)$$

where $n_1$ = number of presences, and $n_0$ = number of absences of the item to be modelled.

### C. Independent variables: Primate zoogeography

1. Initial data: IUCN range maps of the American and African non-human primates.
2. For every continent, building of a matrix of presence (1) / absence (0) of every primate species (columns) in every hexagon (rows): "primate_data"
3. Hierarchical classification of the species ranges, separately for every continent, through the analysis of the 1/0 matrix using the Baroni-Urbani & Buser´s similarity index and the UPGMA classification method.
4. Chorotype variables: Definition of chorotypes through the evaluation of the statistical significance of all clusters in the hierarchical classification using RMacoqui 1.0 software (http://rmacoqui.r-forge.r-project.org/). For every chorotype, quantification, in each hexagon, of the number of species belonging to each chorotype.

**R script for the package "RMacoqui" (https://rmacoqui.r-forge.r-project.org/)**

```
## The data set was a presences/absences matrix for primates of a continent.

data(primate_data)
macoquires <- macoqui(primate_data)

## Friendly 'macoqui' results. Output of interest: "Chorotype Report".

ver.matRmacoqui(macoquires)

## Parameters for chorotype variable quantification. Output of interest: "Chorotypes in
Localities". The SR (species richness) result was used.

locs <- locCorot(macoquires)
ver.matRmacoqui(locs)
```

5. Logistic regression: The objective is to choose the set of chorotype variables to be considered henceforth. The study area is global, and so both African and American chorotypes are included in the analysis.
   a. Dependent variable: Presences (1) and absences (0) of yellow fever cases
   b. Independent variables: African and American chorotype variables.
   c. Selection of independent variables: Conditional forward-backward stepwise procedure.
   d. Estimation of variable coefficients: machine-learning iterative process based on the maximum log-likelihood criterion.
   e. Only the chorotype variables selected by the stepwise logistic regression were considered to be "primate zoogeography" variables henceforth.

**SPSS syntax for the logistic regression**

```
LOGISTIC REGRESSION VARIABLES Dependent_variable
  /METHOD=FSTEP(COND) List_of_chorotype_variables
  /SAVE=PRED
  /CRITERIA=PIN(.05) POUT(.10) ITERATE(20) CUT(.5).
```

## D. Vector models

### D1. 20th-century model for urban vector species *Aedes aegypti* and *Ae. albopictus*

1. Control of the Type I error using the False Discovery Rate (FDR):
   a. RAO´s score test of all environmental variables (respect to the presences and absences of the mosquito species between 1970 and 2000) to get the *p* values.
   b. Pre-selection of variables for which $p < i \times q/v$ [$i$ = position of the variable, arranged in increasing *p* order; $q$ = 0.05; $v$ = total number of environmental variables]. Only these pre-selected variables are considered henceforth.
2. Logistic regression: It provides a probability value ($P_{um20}$), based on environment and space variables, of the urban mosquito (*um*) species to occur in each hexagon during the late 20th century.
   a. Dependent variable: Presences (1) and absences (0) of the mosquito species between 1970 and 2000. *Ae. aegypti* and *Ae. albopictus* are modelled separately.
   b. Independent variables: environment (**A**) and space (**B**) variables.
   c. Selection of independent variables: Conditional forward-backward stepwise procedure.
   d. Estimation of variable coefficients: machine-learning iterative process based on the maximum log-likelihood criterion.
   e. Model evaluation: Hosmer & Lemeshow's goodness of fit.

**SPSS syntax for the logistic regression**
LOGISTIC REGRESSION VARIABLES *Dependent_variable*
/METHOD=FSTEP(COND) *List_of_environment_and_space_variables*
/SAVE=PRED
/CRITERIA=PIN(.05) POUT(.10) ITERATE(20) CUT(.5).

3. Control for excessive multicollinearity:
   a. Identification of variables with Spearman correlation coefficient > 0.8 among the variables selected by the logistic regression.
   b. This being the case, deletion of the least significant correlated variable according to the RAO´s score test, and repetition of the logistic regression.
4. Favourability ($F_{um20}$) values are calculated, based on the probability ($P_{um20}$) values provided by the logistic regression:

$$F_{um20} = \frac{P_{um20}}{1 - P_{um20}} / \left(\frac{n_1}{n_0} + \frac{P_{um20}}{1 - P_{um20}}\right)$$

   where $n_1$ = number of presences, and $n_0$ = number of absences of the modelled mosquito species

### D2. 21st-century model for urban vector species *Aedes aegypti* and *Ae. albopictus*

1. Control of the Type I error using the False Discovery Rate (FDR):
   a. RAO´s score test of all environmental variables (respect to the presences and absences of the mosquito species between 2001 and 2017) to get the *p* values.
   b. Pre-selection of variables for which $p < i \times q/v$ [$i$ = position of the variable, when arranged in increasing $p$ order; $q$ = 0.05; $v$ = total number of environmental variables]. Only these pre-selected variables are considered henceforth.
2. Logistic regression: It provides a probability value ($P_{um21}$), based on environmental and spatial variables, of the urban mosquito (*um*) species to occur in each hexagon during the early 21st century.
   a. Dependent variable: Presences (1) and absences (0) of the mosquito species between 2001 and 2017. *Ae. aegypti* and *Ae. albopictus* are modelled separately.
   b. Independent variables: environment (**A**) and space (**B**) variables, and the “updating variable”, that is the value for the logit equation of this mosquito’s 20th-century model (**D1**). The logit equation is the linear combination of variables selected in section D1.2c (see above).
   c. Selection of independent variables: A two-blocks approach is employed. The updating variable is forced to enter in the model in the first block; environment and space variables are selected in the second block using a conditional forward-backward stepwise procedure.
   d. Estimation of variable coefficients: machine-learning iterative process based on the maximum log-likelihood criterion.
   e. Model evaluation: Hosmer & Lemeshow’s goodness of fit.

**SPSS syntax for the logistic regression**
LOGISTIC REGRESSION VARIABLES *Dependent_variable*
/METHOD=ENTER *20th-century_model_logit*
/METHOD=FSTEP(COND) *List_of_environment_and_space_variables*
/SAVE=PRED
/CRITERIA=PIN(.05) POUT(.10) ITERATE(20) CUT(.5).

3. Control for excessive multicollinearity:
    a. Identification of variables with Spearman correlation coefficient > 0.8 among the variables selected.
    b. This being the case, deletion of the least significant correlated variable according to the RAO´s score test, and repetition of the logistic regression.
4. Favourability ($F_{um21}$) values are calculated, based on the probability ($P_{um21}$) values provided by the logistic regression:

$$F_{um21} = \frac{P_{um21}}{1 - P_{um21}} / \left(\frac{n_1}{n_0} + \frac{P_{um21}}{1 - P_{um21}}\right)$$

where $n_1$ = number of presences, and $n_0$ = number of absences of the modelled mosquito species.

**D3. Model for every sylvatic vector species**

1. Control of the Type I error using the False Discovery Rate (FDR):
    a. RAO´s score test of all environmental variables (respect to the presences and absences of the mosquito species between 1970 and 2017) to get the *p* values.
    b. Pre-selection of variables for which $p < i \times q/v$ [$i$ = position of the variable, when arranged in increasing $p$ order; $q$ = 0.05; $v$ = total number of environmental variables]. Only these pre-selected variables are considered henceforth.
3. Logistic regression: It provides a probability value ($P_{sm}$), based on environmental and spatial variables, of the sylvatic mosquito (*sm*) species to occur in each hexagon
    a. Dependent variable: Presences (1) and absences (0) of the mosquito species between 1970 and 2017. *Haemagogus janthinomys*, *H. leucocelaenus, Sabethes chloropterus*, *Ae. africanus*, and *Ae. vittatus* are modelled separately.
    b. Independent variables: environment (**A**) and space (**B**) variables.
    c. Selection of independent variables: Conditional forward-backward stepwise procedure.
    d. Estimation of variable coefficients: machine-learning iterative process based on the maximum log-likelihood criterion.
    e. Model evaluation: Hosmer & Lemeshow's goodness of fit.

**SPSS syntax for the logistic regression**

```
LOGISTIC REGRESSION VARIABLES Dependent_variable
/METHOD=FSTEP(COND) List_of_environment_and_space_variables
/SAVE=PRED
/CRITERIA=PIN(.05) POUT(.10) ITERATE(20) CUT(.5).
```

4. Control for excessive multicollinearity:
    a. Identification of variables with Spearman correlation coefficient > 0.8 among the variables selected.
    b. This being the case, deletion of the least significant correlated variable according to the RAO´s score test, and repetition of the logistic regression.
5. Favourability ($F_{sm}$) values are calculated, based on the probability ($P_{sm}$) values provided by the logistic regression:

$$F_{\mathrm{sm}} = \frac{P_{sm}}{1 - P_{sm}} / \left(\frac{n_1}{n_0} + \frac{P_{sm}}{1 - P_{sm}}\right)$$

where $n_1$ = number of presences, and $n_0$ = number of absences of the modelled mosquito species.

### D4. Integration of individual vector models using fuzzy logic

1. **20th-century vector model:** Application of the fuzzy union (U) between the models of the seven mosquito species considered (**D1** and **D3**); i.e., in each hexagon, the maximum value shown by any of the mosquito species models is selected. The 20th-century models are used for *Ae. aegypti* and *Ae albopictus*.

**SPSS syntax for fuzzy union between mosquito models, late 20th century**

```
COMPUTE 20_century_vector_model =MAX(Fum20_Ae_aegypti,
Fum20_Ae_albopictus, Fsm_H_janthinomys, Fsm_H_leucocelaenus,
Fsm_S_chloropterus, Fsm_Ae_africanus, Fsm_Ae_vittatus).
EXECUTE.
```

2. **21st-century vector model:** Application of the fuzzy union (U) between the models of the seven mosquito species considered (**D2** and **D3**); i.e., in each hexagon, the maximum value shown by any of the mosquito species models is selected. The 21st-century models are used for *Ae. aegypti* and *Ae albopictus*.

**SPSS syntax for fuzzy union between mosquito models, early 21st century**

```
COMPUTE 21_century_vector_model =MAX(Fum21_Ae_aegypti,
Fum21_Ae_albopictus, Fsm_H_janthinomys, Fsm_H_leucocelaenus,
Fsm_S_chloropterus, Fsm_Ae_africanus, Fsm_Ae_vittatus).
EXECUTE.
```

## E. Baseline disease models:

### E.1. 20th-century disease model

1. Control of the Type I error using the False Discovery Rate (FDR):
   a. RAO´s score test of all environmental variables (respect to the presences and absences of yellow fever cases between 1970 and 2000) to get the *p* values.
   b. Pre-selection of variables for which $p < i \times q/v$ [$i$ = position of the variable, when arranged in increasing $p$ order; $q$ = 0.05; $v$ = total number of environmental variables]. Only these pre-selected variables are considered henceforth.
2. Logistic regression: It provides a probability value ($P_{dis20}$) based on environmental and spatial variables, of yellow fever disease cases (*dis*) to occur in each hexagon during the late 20th century.
   a. Dependent variable: Presences (1) and absences (0) of yellow fever cases between 1970 and 2000.
   b. Independent variables: environment (**A**), space (**B**) and primate zoogeography (**C**) variables.
   c. Selection of independent variables: A two-blocks approach is employed. Environment and space variables are selected the first block; primate zoogeography variables are selected the second block. For this selection, a conditional forward-backward stepwise procedure is used.
   d. Estimation of variable coefficients: machine-learning iterative process based on the maximum log-likelihood criterion.
   e. Model evaluation: Hosmer & Lemeshow's goodness of fit.

**SPSS syntax for the logistic regression**

```
LOGISTIC REGRESSION VARIABLES Dependent_variable
/METHOD=FSTEP(COND) List_of_environment_and_space_variables
/METHOD=FSTEP(COND) Primate_zoogeography_variables
/SAVE=PRED
/CRITERIA=PIN(.05) POUT(.10) ITERATE(20) CUT(.5).
```

3. Control for excessive multicollinearity:

a. Identification of variables with Spearman correlation coefficient > 0.8 among the variables selected.
b. This being the case, deletion of the least significant correlated variable according to the RAO´s score test, and repetition of the logistic regression.

4. Favourability ($F_{dis20}$) values are calculated, based on the probability ($P_{dis20}$) values provided by the logistic regression:

$$F_{dis20} = \frac{P_{dis20}}{1 - P_{dis20}} / \left(\frac{n_1}{n_0} + \frac{P_{dis20}}{1 - P_{dis20}}\right)$$

where $n_1$ = number of presences, and $n_0$ = number of absences of yellow fever cases during the late 20th century.

## E.2. 21st-century disease model

1. Control of the Type I error using the False Discovery Rate (FDR):
   a. RAO´s score test of all environmental variables (respect to the presences and absences of yellow fever cases between 2001 and 2017) to get the *p* values.
   b. Pre-selection of variables for which $p < i \times q/v$ [$i$ = position of the variable, when arranged in increasing $p$ order; $q$ = 0.05; $v$ = total number of environmental variables]. Only these pre-selected variables are considered henceforth.
2. Logistic regression: It provides a probability value ($P_{dis21}$) based on environmental and spatial variables, of yellow fever disease cases (*dis*) to occur in each hexagon during the late 21st century.
   a. Dependent variable: Presences (1) and absences (0) of yellow fever cases between 2001 and 2017.
   b. Independent variables: environmental variables (**A**), spatial variables (**B**), primate zoogeography (**C**) variables, and the "updating variable", that is the value for the logit equation of the 20th-century disease model (**E1**). The logit equation is the linear combination of variables selected in section E1.2c (see above).
   c. Selection of independent variables: A three-blocks approach is employed. The updating variable is forced to enter in the model in the first block; environment and space variables are selected in the second block; primate zoogeography variables are selected in the third block. For this selection, a conditional forward-backward stepwise procedure is used.
   d. Estimation of variable coefficients: machine-learning iterative process based on the maximum log-likelihood criterion.
   e. Model evaluation: Hosmer & Lemeshow's goodness of fit.

**SPSS syntax for the logistic regression**

```
LOGISTIC REGRESSION VARIABLES Dependent_variable
/METHOD=ENTER 20_century_disease_model_logit
/METHOD=FSTEP(COND) List_of_environment_and_space_variables
/METHOD=FSTEP(COND) Primate_zoogeography_variables
/SAVE=PRED
/CRITERIA=PIN(.05) POUT(.10) ITERATE(20) CUT(.5).
```

5. Control for excessive multicollinearity:
   a. Identification of variables with Spearman correlation coefficient > 0.8 among the variables selected.
   b. This being the case, deletion of the least significant correlated variable according to the RAO´s score test, and repetition of the logistic regression.
6. Computation of favourability ($F_{dis21}$) values based on the probability ($P_{dis21}$) values provided by the logistic regression:

$$F_{dis21} = \frac{P_{dis21}}{1 - P_{dis21}} / \left(\frac{n_1}{n_0} + \frac{P_{dis21}}{1 - P_{dis21}}\right)$$

where $n_1$ = number of presences, and $n_0$ = number of absences of yellow fever cases during the early 21st century.

**F. Transmission risk models**:

1. **20th-century transmission risk model:** Application of the fuzzy intersection (∩) between the 20th-century vector model (**D4.1**) and the 20th-century disease model (**E1**); i.e., in each hexagon, the minimum value shown by any of these models is selected.

**SPSS syntax for fuzzy intersection between the vector and disease models, late 20th century**

```
COMPUTE 20_century_transmission_model=MIN(20_century_vector_model,
20_century_disease_model).
EXECUTE.
```

2. **21st-century transmission risk model:** Application of the fuzzy intersection (∩) between the 21st-century vector model (**D4.2**) and the 21st -century disease model (**E2**); i.e., in each hexagon, the minimum value shown by any of these models is selected.

**SPSS syntax for fuzzy intersection between the vector and disease models, late 21st century**

```
COMPUTE 21_century_transmission_model=MIN(21_century_vector_model,
21_century_disease_model).
EXECUTE.
```
