# Supplementary material for: Yellow fever surveillance suggests zoonotic and anthroponotic emergent potential
Source: Commun Biol. 2022 Jun 2;5:530. doi: 10.1038/s42003-022-03492-9 (PMC9163115; doi:10.1038/s42003-022-03492-9)
Supplement: Supplementary file 3 — Description of Additional Supplementary Files [file 42003_2022_3492_MOESM3_ESM.pdf]

Description of Additional Supplementary Files

File name: Supplementary Data 1
Description: List of sources of yellow fever case reports for the period 1970-2020.

File name: Supplementary Data 2
Description: Occurrence of yellow fever case reports in the spatial units used for modelling (i.e., in 7,774-km2 hexagons). Geographic coordinates of hexagon centroids are shown.
