# Supplementary material for: Yellow fever surveillance suggests zoonotic and anthroponotic emergent potential
Source: Commun Biol. 2022 Jun 2;5:530. doi: 10.1038/s42003-022-03492-9 (PMC9163115; doi:10.1038/s42003-022-03492-9)
Supplement: Supplementary file 6 — Reporting Summary [file 42003_2022_3492_MOESM6_ESM.pdf]

nature portfolio

Corresponding author(s): Alisa Aliaga-Samanez

Last updated by author(s): Apr 22, 2022

Reporting Summary

Nature Portfolio wishes to improve the reproducibility of the work that we publish. This form provides structure for consistency and transparency in reporting. For further information on Nature Portfolio policies, see our Editorial Policies and the Editorial Policy Checklist.

Statistics

For all statistical analyses, confirm that the following items are present in the figure legend, table legend, main text, or Methods section.

n/a	Confirmed	
☐	☒	The exact sample size (*n*) for each experimental group/condition, given as a discrete number and unit of measurement
☒	☐	A statement on whether measurements were taken from distinct samples or whether the same sample was measured repeatedly
☐	☒	The statistical test(s) used AND whether they are one- or two-sided<br>*Only common tests should be described solely by name; describe more complex techniques in the Methods section.*
☐	☒	A description of all covariates tested
☒	☐	A description of any assumptions or corrections, such as tests of normality and adjustment for multiple comparisons
☐	☒	A full description of the statistical parameters including central tendency (e.g. means) or other basic estimates (e.g. regression coefficient) AND variation (e.g. standard deviation) or associated estimates of uncertainty (e.g. confidence intervals)
☐	☒	For null hypothesis testing, the test statistic (e.g. *F*, *t*, *r*) with confidence intervals, effect sizes, degrees of freedom and *P* value noted<br>*Give P values as exact values whenever suitable.*
☒	☐	For Bayesian analysis, information on the choice of priors and Markov chain Monte Carlo settings
☒	☐	For hierarchical and complex designs, identification of the appropriate level for tests and full reporting of outcomes
☐	☒	Estimates of effect sizes (e.g. Cohen's *d*, Pearson's *r*), indicating how they were calculated

Our web collection on statistics for biologists contains articles on many of the points above.

Software and code

Policy information about availability of computer code

Data collection	We took georreferenced yellow-fever cases for the period 1970–2016 from Shearer et al. (Shearer, F. M. et al. Lancet Glob. Heal. 6, e270–e278 (2018) (6)). We extracted additional cases for the period 1970–2020 from various sources available online (see Supplementary data 1), including various sources detailed below in the "study design" section. The georeferenced presences of vectors involved in the urban cycle of the yellow fever (i.e., the mosquito species Ae. aegypti and Ae. albopictus) were taken from “The global compendium of the Ae. aegypti and Ae. Albopictus occurrence” for the period 1970–2014. We complemented these records with georeferenced data scientifically validated for the period 2014–2017, taken from VectorBase (https://www.vectorbase.org/) and Mosquito Alert (http://www.mosquitoalert.com/). We included both species because, although Ae. Aegypti is the main vector of yellow fever, Ae. albopictus can also transmit the yellow fever virus to humans (Couto-Lima, Di. et al. Sci. Rep. 7, 1–12 (2017) (4); Amraoui, F. et al. Sci. Rep. 8, 14337 (2018)(52)). Distribution maps of non-human primates were obtained from the IUCN for South-America and Africa. The final data set of yellow fever cases and vector occurrences were transformed into a binary variable per study period representing the presence or absence of occurrences in each hexagon, using ArcMap 10.7. In the case of primates, in each hexagon, the number of species belonging to each chorotype was quantified using RMacoqui 1.0 software (http://rmacoqui.r-forge.r-project.org/). We used zonal statistic tools to calculate average enviromental/spatial variable values using ArcMap 10.7.
Data analysis	We latticed the data using a worldwide grid composed of 18,874 hexagonal 7,774-km2 units, built using Discrete Global for R (https://github.com/r-barnes/dggridR). We defined primate chorotypes and evaluated their statistical significance using RMacoqui 1.0 software (http://rmacoqui.r-forge.r-project.org/). We used IBM-SPSS Statistics 24 software to perform the models and all the associated tests. We used ArcMap 10.7 to visualise the cartographic models.

For manuscripts utilizing custom algorithms or software that are central to the research but not yet described in published literature, software must be made available to editors and reviewers. We strongly encourage code deposition in a community repository (e.g. GitHub). See the Nature Portfolio guidelines for submitting code & software for further information.

Data

Policy information about availability of data

All manuscripts must include a data availability statement. This statement should provide the following information, where applicable:

- Accession codes, unique identifiers, or web links for publicly available datasets
- A description of any restrictions on data availability
- For clinical datasets or third party data, please ensure that the statement adheres to our policy

The sources for all data supporting the results of this study are cited in the main text, in Supplementary table 3, and in Supplementary data 1. The occurrence of yellow fever case reports in the spatial units employed here can be found in Supplementary data 2.

Field-specific reporting

Please select the one below that is the best fit for your research. If you are not sure, read the appropriate sections before making your selection.

☐ Life sciences ☐ Behavioural & social sciences ☒ Ecological, evolutionary & environmental sciences

For a reference copy of the document with all sections, see nature.com/documents/nr-reporting-summary-flat.pdf

Ecological, evolutionary & environmental sciences study design

All studies must disclose on these points even when the disclosure is negative.

Study description	We assess where the risk of yellow fever infections in humans could be being amplified by the contribution of enzootic cycles based on primate assemblages and a pool of sylvatic mosquito vectors. Given the current context of yellow fever geographic spread, we address this challenge through a temporal stratification of disease cases, thus assuming that the current risk is a combination of past trends and the arrival of new factors.
Research sample	We used worldwide georeferenced cases of yellow fever in humans for a period of 51 years (from 1970 to 2020). We took georreferenced yellow fever cases for the period 1970–2016 from Shearer et al. (Shearer, F. M. et al. Lancet Glob. Heal. 6, e270–e278 (2018) (6)). We extracted additional cases for the period 1970–2020 from various sources (see Supplementary data 1). The georeferenced presences of vectors involved in the urban cycle of the yellow fever (i.e., the mosquitoes Ae. aegypti and Ae. albopictus) were taken from “The global compendium of the Ae. aegypti and Ae. Albopictus occurrence” (Kraemer, M. U. G. et al. Sci. Data 2, 1–8 (2015)(26)) for the period 1970–2014. We complemented these records with georeferenced data scientifically validated for the period 2014–2017, taken from VectorBase (https://www.vectorbase.org/) and Mosquito Alert (http://www.mosquitoalert.com/). In addition, we included georeferenced occurrence data of sylvatic vectors (Haemagogus janthinomys, H. leucocelaenus and Sabethes chloropterus in South America; Ae. africanus and Ae. vittatus in Africa), which were obtained from Vectormap (vectormap.si.edu) and Gbif (https://gbif.org). Distribution maps of non-human primates were obtained from the IUCN for South-America and Africa.
Sampling strategy	This research was made with all the information available worldwide on yellow fever case reports, and vector mosquito occurrences. We made no sampling ourselves, and the above section "research sample" exposes how we made our data set.
Data collection	All spatially explicit data (i.e., yellow fever case records, mosquito occurrences, primate ranges, environmental variables) were projected onto a worldwide grid composed of 18,874 hexagonal units of 7,774 km2. We took georreferenced yellow-fever cases for the period 1970–2016 from Shearer et al. 2018 (Shearer, F. M. et al. Lancet Glob. Heal. 6, e270–e278 (2018) (6)). We extracted additional cases for the period 1970–2020 from various sources (Supplementary data 1), including: ProMED-mail: Program of International society for infectious diseases; World Health Organization (WHO): Yellow fever outbreak weekly situation reports, Rapport de situation fievre jaune en RD Congo and Weekly epidemiological record; Health Ministry of different countries: Epidemiological Bulletins of yellow fever in Brazil, Peru, Colombia, and Paraguay; Pan American Health Organization (PAHO): Epidemiological Update Yellow Fever; European Centre for Disease Prevention and Control (ECDC): Communicable disease threats report and Rapid risk assessment report; Nigeria Centre for Disease Control (NCDC): Situation report, yellow fever outbreak in Nigeria and Global Infectious Disease and Epidemiology Online Network (GIDEON). The reported cases were complemented with publications available since 2016 with geo-referenced information on case location (Supplementary data 1). In addition, information was also sought on cases reported in French and Portuguese from local news reports in Africa.<br>The georeferenced presences of vectors involved in the urban cycle of the yellow fever (i.e., the mosquitoes Aedes aegypti and Ae. albopictus) were taken from “The global compendium of the Aedes aegypti and Ae. Albopictus occurrence” (Kraemer, M. U. G. et al. Sci. Data 2, 1–8 (2015)(26)) for the period 1970–2014. We complemented these records with georeferenced data scientifically validated for the period 2014–2017, taken from VectorBase (https://www.vectorbase.org/) and Mosquito Alert (http://www.mosquitoalert.com/). In addition, we included georeferenced occurrence data of sylvatic vectors which were obtained from Vectormap (vectormap.si.edu) and Gbif (https://gbif.org). Distribution maps of non-human primates were obtained from the IUCN for South-America and Africa. We represented in the hexagonal lattice the reported cases of yellow fever or presences of vectors that had a precise location or that were referred to administrative unit smaller than or of similar size to the hexagons. This database was transformed into a binary variable per study period representing the presence or absence of reported cases of yellow fever in each hexagon. In the case of primates, in each hexagon, the number of species belonging to each chorotype was quantified.
Timing and spatial scale	We used georeferenced cases of yellow fever in humans for a period of 51 years (from 1970 to 2020) at the global scale. All spatially explicit data were projected onto a worldwide grid composed of 18,874 hexagonal units of 7,774 km2. This study period starts immediately after the suspension of the use of DDT due to to the appearance of resistance of Ae. aegypti in the late 1960s in several

countries, after 50 years of eradication efforts. The temporal extent for analysis purposes was divided into three periods: 1970–2000 ("the late 20th century"), 2001–2017 ("the early 21st century"), and 2018–2020. Although the limit between periods at the turn of the century is arbitrary, it reflects: 1) Distributional changes in the ranges of the Ae. aegypti and Ae. albopictus vectors (Liu-Helmersson, J., Brännström, Å., Sewe, M. O., Semenza, J. C. & Rocklöv, J. Front. Public Heal. 7, (2019)(51); 2) after 1999, the yellow fever genotype I has spread outside the endemic regions, and the genotype I modern-lineage has caused all major yellow fever outbreaks detected in non-endemic regions of South America since 2000 (Mir, D. et al. Sci. Rep. 7, 1–9 (2017)(13)); 3) the maximum potential of globalization was realised at the beginning of the 21st century with the opening of international borders, the widespread access to the Internet and to cell phones, and the generalization of online travel booking and of low-cost flights (Aliaga-Samanez, A. et al. PLoS Negl. Trop. Dis. 15, e0009496 (2021)(34)).
Given the current context of yellow fever geographic spread, we address this challenge through a temporal stratification of disease cases, thus assuming that the current risk is a combination of past trends and the arrival of new factors. For this aim, we made a model for the late 20th century (1970-2000), and another for the early 21st century (2001-2017). Predictions afforded by the late 20th century model were validated using the distribution of disease cases in the early 21st century, and prediction validations for the early 21st-century-model were addressed with post-2017 case records.

Data exclusions

We only excluded, from the global yellow fever information available, the cases for which a suitable georreferenciation was not provided, given the spatial resolution of our research (i.e., we only used the reported cases that had a precise location, or those referred to administrative unit smaller than or of similar size to the 7,774-km2 hexagons).

Reproducibility

This is an observational study using many years of survey data and can be replicated.

Randomization

Randomization was not relevant to this study. This study analysis is an observational mapping and there were no experimental groups.

Blinding

Blinding was not relevant to this study, as it was an observational study using survey and report data

Did the study involve field work? ☐ Yes ☒ No

Reporting for specific materials, systems and methods

We require information from authors about some types of materials, experimental systems and methods used in many studies. Here, indicate whether each material, system or method listed is relevant to your study. If you are not sure if a list item applies to your research, read the appropriate section before selecting a response.

Materials & experimental systems

n/a	Involved in the study	
☒	☐	Antibodies
☒	☐	Eukaryotic cell lines
☒	☐	Palaeontology and archaeology
☒	☐	Animals and other organisms
☒	☐	Human research participants
☒	☐	Clinical data
☒	☐	Dual use research of concern

Methods

n/a	Involved in the study	
☒	☐	ChIP-seq
☒	☐	Flow cytometry
☒	☐	MRI-based neuroimaging
